# Functional decline at 1 year in hospitalized elderly pneumonia with SARS‐CoV‐2 Omicron variant: Comparison with the ancestral strain and Alpha variant

**DOI:** 10.1111/irv.13251

**Published:** 2024-02-07

**Authors:** Naoyuki Miyashita, Yasushi Nakamori, Makoto Ogata, Naoki Fukuda, Akihisa Yamura, Tomoki Ito

**Affiliations:** ^1^ First Department of Internal Medicine, Division of Respiratory Medicine, Infectious Disease and Allergology Kansai Medical University Hirakata Japan; ^2^ Department of Emergency Medicine Kansai Medical University Medical Center Moriguchi Japan

**Keywords:** activity of daily living, COVID‐19, functional outcome, Omicron variant, SARS‐CoV‐2

## Abstract

In the SARS‐CoV‐2 Omicron period, the pattern of pneumonia changed from primarily viral pneumonia to pneumonia mixed with bacteria in the elderly. We investigated functional outcomes at 1 year after hospital discharge in patients with primary Omicron pneumonia and pneumonia mixed with bacteria, mainly aspiration pneumonia. Functional decline rates calculated using the Barthel Index at 1 year after hospital discharge were significantly higher in the pneumonia mixed with bacteria group than the primary viral pneumonia group (42.6% vs. 20.5%, *p* < 0.0001). It is necessary to consider early rehabilitation and treatment in elderly patients even when the predominant strain is the Omicron variant.

## 1 INTRODUCTION

Functional decline during or after hospitalization is associated with adverse health outcomes, prolonged hospital stays due to more frequent occurrences of hospital complications, and more frequent episodes of early hospital admission.^1^, ^2^, ^3^, ^4^ In a previous study, we demonstrated that advanced age was a risk factor for functional decline at 1 year in hospitalized elderly patients with pneumonia due to the SARS‐CoV‐2 ancestral strain and Alpha variant.^5^ These variants of SARS‐CoV‐2 were replaced by the Omicron variant, which exhibited reduced pathogenicity compared with the ancestral strain, Alpha variant, and Delta variant.^6^ In addition, vaccination against SARS‐CoV‐2 progressed, and the mortality rate dropped markedly.^7^ During the period when the ancestral strain, Alpha variant, and Delta variant were dominant, bacterial coinfection and secondary bacterial infection in patients with COVID‐19 were low.^8^, ^9^, ^10^ In the Omicron period, however, the pattern of pneumonia changed from primarily viral pneumonia to pneumonia mixed with bacteria, mainly aspiration pneumonia.

The Japan Respiratory Society (JRS) pneumonia guidelines emphasize the importance of pneumonia prevention rather than antibiotic therapy to avoid deterioration of physical function in the elderly.^11^ Functional decline after hospitalization due to pneumonia often induces aspiration pneumonia, especially in those aged ≥80 years old after acute febrile illness such as a viral infection.^11^ The objective of this study was to clarify functional outcomes at 1 year after hospital discharge in elderly patients (≥80 years old) hospitalized for pneumonia due to the SARS‐CoV‐2 Omicron variant.

## 2 PATIENTS AND METHODS

### 2.1 Study populations

The present study was conducted at five institutions (Kansai Medical University Hospital, Kansai Medical University Medical Center, Kansai Medical University Kori Hospital, Kansai Medical University Kuzuha Hospital, and Kansai Medical University Temmabashi General Clinic) between December 2021 and August 2022. COVID‐19 was diagnosed with positive reverse transcription polymerase chain reaction (RT‐PCR) results from sputum or nasopharyngeal swab specimens in accordance with the protocol recommended by the National Institute of Infectious Diseases, Japan. Identification of SARS‐CoV‐2 variants was performed by Sanger sequencing of the Spike coding gene using an ABI 3500 analyzer (Applied Biosystems).^12^ Informed consent was obtained from all patients, and the study protocol was approved by the Ethics Committee of Kansai Medical University (approval number 2020319).

### 2.2 Evaluation of functional outcomes

Activities of daily living (ADL) was used to calculate the Barthel Index, which consists of the following 10 indices: feeding; bathing; grooming; dressing; bowels; bladder; toilet use; transfers; morbidity; and stairs.^13^ In the present study, attending physicians calculated the difference in ADL scores using a questionnaire between baseline on admission (confirmed 1 week before admission as baseline), at hospital discharge, and 1 year after discharge from our hospitals. The difference was categorized into two groups: declined (≥1) and not declined (0).

### 2.3 Pneumonia case enrollment

We enrolled adult patients consecutively diagnosed with pneumonia who were SARS‐CoV‐2 RT‐PCR positive. The diagnosis of pneumonia was based on clinical signs and symptoms (cough, fever, productive sputum, dyspnea, or chest pain) and radiographic pulmonary abnormalities that were at least segmental and were not as a result of preexisting or other known causes. Exclusion criteria included the following: immunosuppressive illness (i.e., HIV positive, neutropenia secondary to chemotherapy, use of >20 mg/day prednisone or other immunosuppressive agents, and history of organ transplant) and active tuberculosis. All cases of pneumonia occurring more than 3 days after hospitalization were considered nosocomial and were excluded.

The total scores of the Barthel Index range from 0 to 100. High scores indicate better ADL and less than 40 points indicate full assistance. In this study, thus, patients with full assistance and cognitive decline were excluded because it is difficult to evaluate the ADL.

### 2.4 Definition of bacterial coinfection and secondary bacterial pneumonia

When the RT‐PCR for SARS‐CoV‐2 was positive, microbiological tests, such as Gram stain, cultures, urinary antigen tests and serological tests, were performed as described previously.^14^ Omicron pneumonia with mixed bacteria was defined if one of the following conditions was present: (1) blood or pleural fluid cultures yielding the presence of bacterial pathogen; (2) urinary antigen test results positive for *Legionella pneumophila* or *Streptococcus pneumoniae*; (3) respiratory specimen culture results positive for *Mycoplasma pneumoniae*, *Chlamydia*, or *Legionella* species; (4) a fourfold increase in the antibody titer for *M. pneumoniae*, *Legionella* species, *Coxiella burnetii*, or *Chlamydia* species (IgM or IgG) using the paired serum for at least 2 weeks; (5) an organism showing heavy (≥10^7^ cfu/mL) or moderate (10^6^ cfu/mL) growth of a predominant bacterium on a sputum culture in combination with Gram stain findings; or (6) bacterial pneumonia prediction score 0 or 1 point using the JRS pneumonia guidelines.^15^

Aspiration pneumonia was defined according to the Japanese Study Group on Aspiration Pulmonary Disease definition as pneumonia in a patient with a predisposition to aspiration because of dysphagia or swallowing disorders.^11^ Swallowing function was assessed using the water swallowing test, repetitive saliva swallowing test, simple‐swallowing provocation test, and video fluorography.^11^ When swallowing function was not assessed using these examinations, the presence of overt symptoms of dysphagia or a medical history of aspiration was determined as a swallowing disorder in the patient.

### 2.5 Statistical analysis

Statistical analysis was performed using Stat View version 5.0. (SAS Institute Inc, Cary, NC, USA). Percentage of patients with male and female, Omicron subvariants, comorbid illnesses, SARS‐CoV‐2 mRNA vaccination, prior SARS‐CoV‐2 infection, deterioration of physical activity, and in‐hospital mortality was analyzed using Fisher's exact test. Age was compared using Student's *t*‐test and Barthel Index was compared using Mann–Whitney *U* test.

## 3 RESULTS

### 3.1 Patient characteristics

During the study period, 891 elderly patients with pneumonia due to SARS‐CoV‐2 Omicron variant were recognized. Of these, we enrolled 303 patients with primary viral pneumonia and 326 patients with pneumonia mixed with bacteria (mainly aspiration pneumonia) that we could follow‐up for 1 year (Table 1). Of the patients with primary viral pneumonia, 112 cases were the Omicron BA.1 subvariant, 70 cases were the Omicron BA.2 subvariant, and 121 cases were the Omicron BA.5 subvariant. Of patients with pneumonia mixed with bacteria, 118 cases were the Omicron BA.1 subvariant, 72 cases were the Omicron BA.2 subvariant, and 136 cases were the Omicron BA.5 subvariant. Percentage of the subvariant strains was the same between two groups. Vaccination status was also the same between two groups.

**TABLE 1:** Clinical characteristics and outcomes of elderly pneumonia with SARS‐CoV‐2 Omicron variant.

Variables	Primary viral pneumonia	Pneumonia mixed with bacteria	*p* value
No. of patients	303	326	
Median age (IQR), years	84 (81–86)	84 (81–87)	0.8413
No. of males/females	151/152	168/158	0.6903
No. (%) of patients with Omicron subvariants			
BA.1	112 (37.0)	118 (36.2)	0.8686
BA.2	70 (23.1)	72 (22.1)	0.7753
BA.5	121 (39.9)	136 (41.7)	0.6851
No. (%) of patients with comorbid illnesses			
Diabetes mellitus	82 (27.1)	92 (28.2)	0.7892
Chronic lung disease	72 (23.8)	66 (20.2)	0.2909
Chronic heart disease	62 (20.5)	70 (21.5)	0.7697
Cerebrovascular disease	40 (13.2)	71 (21.8)	0.0063
Chronic renal disease	38 (12.5)	23 (18.5)	0.0220
Neoplastic disease	30 (9.9)	42 (12.9)	0.2608
Autoimmune disease	25 (8.3)	16 (4.9)	0.1061
Chronic liver disease	10 (3.3)	19 (5.8)	0.1821
No. (%) of patients with SARS‐CoV‐2 mRNA vaccination			
Never	52 (17.2)	55 (16.9)	>0.9999
One vaccination	1 (0.3)	0	0.4817
Two vaccinations	149 (49.2)	166 (50.9)	0.6902
Three vaccinations	101 (33.3)	105 (32.2)	0.7989
No. (%) of patients with prior SARS‐CoV‐2 infection	17 (5.6)	19 (5.8)	>0.9999
No. (%) of patients with deterioration of physical activity			
At hospital discharge	122 (40.3)	171 (52.5)	0.0024
At 1 year after hospital discharge	62 (20.5)	139 (42.6)	<0.0001
Barthel Index (average)			
Before admission to hospitals	61	61	>0.9999
At hospital discharge	32	16	<0.0001
At 1 year after hospital discharge	49	27	<0.0001
No. (%) of patients with in‐hospital mortality	28 (9.2)	53 (16.3)	0.0089

*Note*: Continuous values are presented as medians and interquartile ranges (IQRs) and categorical/binary values as counts and percentages.

### 3.2 Functional decline in the Omicron group

Functional decline rates at the time of hospital discharge were higher in the primary viral pneumonia group than the pneumonia mixed with bacteria group (52.3% vs. 40.3%, *p* = 0.0024) (Table 1). Of 171 patients in the pneumonia mixed with bacteria group who had a decline in physical function at the time of hospital discharge, 139 patients (81.3%) still showed functional decline at 1 year later. On the other hand, in the primary viral pneumonia group, 20.5% of patients had functional decline at 1 year after hospital discharge, which is significantly lower (*p* < 0.0001).

### 3.3 Functional decline in the Omicron subvariant groups

Among the BA.1, BA.2, and BA.5 Omicron subvariant groups, functional decline rates at the time of hospital discharge and at 1 year after hospital discharge were similar in primary viral pneumonia group (Table 2) and pneumonia mixed with bacteria group (Table 3).

**TABLE 2:** Functional decline in patients with primary SARS‐CoV‐2 Omicron pneumonia among BA.1, BA.2, and BA.5 subvariants.

Variables	BA.1 subvariant	BA.2 subvariant	BA.5 subvariant
No. of patients	112	70	121
No. (%) of patients with deterioration of physical activity			
At hospital discharge	48 (42.9)	26 (37.1)	48 (39.7)
At 1 year after hospital discharge	24 (21.4)	13 (18.6)	25 (20.7)
Barthel Index (average)			
Before admission to hospitals	63	61	62
At hospital discharge	33	31	32
At 1 year after hospital discharge	49	51	50

*Note*: Categorical/binary values as counts and percentages.

**TABLE 3:** Functional decline in patients with SARS‐CoV‐2 Omicron pneumonia mixed with bacteria among BA.1, BA.2, and BA.5 subvariants*.*

Variables	BA.1 subvariant	BA.2 subvariant	BA.5 subvariant
No. of patients	118	72	136
No. (%) of patients with deterioration of physical activity			
At hospital discharge	63 (53.4)	35 (48.6)	73 (53.7)
At 1 year after hospital discharge	51 (43.2)	30 (41.7)	58 (42.6)
Barthel Index (average)			
Before admission to hospitals	60	62	61
At hospital discharge	16	15	16
At 1 year after hospital discharge	25	29	27

*Note*: Categorical/binary values as counts and percentages.

## 4 DISCUSSION

In a hamster model, the Omicron variant showed decreased lung infectivity and lower pathogenicity than Delta and ancestral SARS‐CoV‐2.^6^ In addition, disease severity was attenuated by immunity from prior SARS‐CoV‐2 infection and vaccination. The intrinsic severity of Omicron and acquired immunity influenced the decline in mortality rates. Global case fatalities ranged from 1.7% to 39.0% in February to March 2020 when the ancestral variant was predominant; however, case fatalities fell to below 0.3% in July to August 2022 when Omicron was predominant in Japan.^7^

In our previous study, we followed up elderly patients aged ≥80 years old with pneumonia due to the SARS‐CoV‐2 ancestral strain and Alpha variant who had not received SARS‐CoV‐2 vaccination and demonstrated that 42.5% showed a decline in function 1 year after hospital discharge compared with their baseline ADL function.^6^ In the present study, functional decline rates at 1 year after hospital discharge were significantly lower in the primary Omicron variant pneumonia group (20.5%) than the primary ancestral strain and Alpha variant pneumonia group (*p* < 0.0001). However, more than 80% patients with Omicron variant pneumonia had received SARS‐CoV‐2 vaccination at least two times. In contrast, functional decline rates at 1 year after hospital discharge were similar between the Omicron pneumonia mixed with bacteria group who received SARS‐CoV‐2 vaccination and the primary ancestral strain and Alpha variant pneumonia group (*p* > 0.9999).

With the decrease in pathogenicity of the Omicron variant and immunity from prior SARS‐CoV‐2 infection and vaccination, functional decline rates at 1 year after hospital discharge were significantly decreased in the primary Omicron variant pneumonia group compared with the primary ancestral strain and Alpha variant pneumonia group. On the other hand, a high incidence of functional decline was observed in elderly patients with Omicron pneumonia with bacterial co‐infection, mainly aspiration pneumonia. However, the comorbid conditions, cerebrovascular disease and chronic renal disease, were significantly more frequent in patients with bacterial coinfection compared with those with primary viral pneumonia group. Thus, it is quite possible that comorbid conditions affect the physical functional decline in elderly COVID‐19 patients with bacterial coinfection.

In conclusion, it is necessary to consider early rehabilitation and treatment in elderly patients even when the predominant strain is the Omicron variant.

## AUTHOR CONTRIBUTIONS

All the authors conceived the study, participated in its design and coordination, and collected and managed the data, including quality control. Naoyuki Miyashita and Yasushi Nakamori drafted the manuscript, and all authors contributed substantially to its revision. All the authors read and approved the final manuscript.

## CONFLICT OF INTEREST STATEMENT

The authors declare that they have no competing interests.

### PEER REVIEW

The peer review history for this article is available at https://www.webofscience.com/api/gateway/wos/peer-review/10.1111/irv.13251.

## ETHICS STATEMENT

The study protocol was approved by the Ethics Committee at Kansai Medical University (approval number 2020319) and all participating facilities. Informed consent was obtained from all individual participants in the study.

## Data Availability

The data will not be shared because of participant confidentiality.
